# Interpersonal style should be included in taxonomies of behavior change techniques

**DOI:** 10.3389/fpsyg.2014.00254

**Published:** 2014-03-25

**Authors:** Martin S. Hagger, Sarah J. Hardcastle

**Affiliations:** ^1^Health Psychology and Behavioural Medicine Research Group, Faculty of Health Sciences, School of Psychology and Speech Pathology, Curtin UniversityPerth, WA, Australia; ^2^School of Sport and Service Management, University of BrightonEastbourne, UK

**Keywords:** health psychology, behavioral medicine, behavior-change interventions, autonomy support, motivational interviewing

Health practitioners and researchers in behavioral medicine recognize the value of interventions aimed at promoting uptake and maintenance of health behavior to prevent chronic illness. Although there have been advances in interventions that promote health behavior change and their effectiveness, gaps in knowledge exist. In particular, the precise components or *techniques* of behavioral interventions that lead to effective behavior change and the mechanisms involved have yet to be fully elucidated. Efforts to identify these techniques have been hindered by a lack of detail in, and systematic reporting of, the content and protocol of behavior-change interventions (Michie and Johnston, 2012). Recent efforts aimed at classifying behavior change techniques (BCTs) have been important in helping to identify the *active ingredients* of behavior-change interventions. This is important for the replication of interventions, identification of the mechanisms involved, and development of a “common language” for BCTs. Michie et al. (2013) present a hierarchically-clustered taxonomy to develop a BCT taxonomy based on consensus and serves as a starting point for the development of future taxonomies. In this article we contend that authors of BCT taxonomies have focused their attention exclusively on intervention content and should pay closer attention to the role *interpersonal style* plays in promoting behavior change. We use two approaches to behavior change that involve both content and interpersonal style to illustrate our point: autonomy support and motivational interviewing. We argue that interpersonal style is a unique set of techniques that likely interact with other content-related BCTs in affecting behavior change.

Michie et al. acknowledge that “mode and context of delivery, and competence of those delivering the intervention would… benefit from being specified by detailed taxonomies” (p.93). Interpersonal style is likely to be encompassed by this caveat, but what is meant by interpersonal style and how it might fit within future taxonomies needs clarification. Interpersonal style should not be equated with mode of delivery. Mode of delivery is the means by which intervention content is communicated to targets or clients such as print communication, audio-visual media, or orally via a health practitioner. Interpersonal style refers to the manner by which intervention content is presented to the target audience and could be delivered by multiple modes. Interpersonal style includes the type of language used in delivering intervention content and interactions between the target audience of the intervention and the health practitioner delivering the intervention. We will illustrate these features of interpersonal style BCTs in the following examples.

Our first example comes from self-determination theory (SDT; Deci and Ryan, 2000). Central to the theory is the premise that individuals will be more likely to initiate and maintain behavior if it is perceived as autonomously motivated. Autonomous motivation means engaging in the behavior to attain *self-determined* outcomes and in the absence of external contingencies. Autonomous motivation can be promoted by social agents through the provision of choice, acknowledging conflict, avoiding controlling language, and external reinforcers, and fostering personally-relevant goals (Hagger et al., 2006, 2007; Hagger and Chatzisarantis, 2009). Some of these techniques relate to content, such as acknowledging conflict, but others depend on social agents adopting the appropriate interpersonal style. For example, avoiding controlling language means refraining from using terms like “should” and “must.” Other intervention techniques outlined in Michie et al.'s taxonomy such as goal setting, threats, and rewards could be delivered in an “autonomy supportive” interpersonal style. In fact, according to SDT, delivering external contingencies like threats and incentives in an autonomy-supportive manner may promote behavioral adherence (Hagger and Chatzisarantis, 2011). This is because autonomy support may illustrate the informational aspect of the incentive and prevent it becoming the “be all and end all” of the behavior. Instead, individuals will view incentives as informing them of their competence and progress. Competence and autonomous motivation therefore reflect the mechanisms, and key mediators, of the effect of an autonomy supportive style on behavior change. An autonomy-supportive interpersonal style may be an additional dimension to behavior-change interventions and interact with other BCTs in affecting behavior change.

Motivational interviewing (MI; Miller and Rollnick, 2013) is our second example. MI is a therapeutic approach to promoting behavior change with multiple techniques that focus on increasing individuals' motivation for behavior change, usually through one-on-one patient-practitioner sessions. MI includes various BCTs some of which have direct equivalents in Michie et al.'s taxonomy such as developing discrepancy between current behavior and goal standard and comparative imagining of future outcomes. However, central to MI is its client-centered focus, often referred to as the “spirit” of MI, which relates to the interpersonal style of the person delivering the MI intervention. For example, MI aims to elicit clients' own reasons for change rather than focus on providing information about change alone (Hardcastle et al., 2012, 2013). The techniques of expressing empathy, rolling with resistance, and promoting client autonomy require the practitioner adopt appropriate language and use a reflective, non-confrontational, non-judgemental, and supportive style to encourage the client to openly explore behavior change options. Some techniques adopted in MI have been identified in BCT taxonomies (Abraham and Michie, 2008; Morton et al., 2014), but the inclusion tends to lack precision and the interpersonal style components have tended to be neglected or omitted. The interpersonal components of MI techniques should, therefore, be incorporated into BCT taxonomies as these are frequently cited as the most efficacious components of MI. An example of an interpersonal style component relevant to SDT and MI that might be coded alongside BCTs is “provision of support and emphasis on autonomy.” This could be evidenced by a practitioner supporting and fostering client choice and exploring options for behavior change as opposed to restricting choice and dictating behavioral decisions to the client.

In conclusion, BCT taxonomies are generally silent on techniques that relate to the interpersonal style of delivery of behavior change intervention content. Future taxonomies should incorporate these components as BCTs in their own right sitting alongside the content-related BCTs. Such an endeavor is essential if the effectiveness of complex interventions that adopt both content and interpersonal style BCTs is to be adequately evaluated.

## Author contributions

Martin S. Hagger and Sarah J. Hardcastle conceived the ideas presented in the article and drafted the article.
